# Supplementary material for: Economic effects of conversion from county (or county-level city) to municipal district in China
Source: PLoS One. 2022 Sep 12;17(9):e0272267. doi: 10.1371/journal.pone.0272267 (PMC9467364; doi:10.1371/journal.pone.0272267)
Supplement: Attachment — Submitted filename: Response to Reviewers.docx [file pone.0272267.s002.docx]

**Revision notes**

Dear Editor:

Thanks to the editorial teachers and external audit experts put forward valuable opinions, so that this paper benefited a lot. According to the comments and suggestions of reviewers, this paper is modified as follows:

**Reviewer #1:**

**1.amendments**: “the INTRODUCTION need to be properly situated by pointing out (i) the justification for the study, (ii) gaps in the literature, (iii) scope justification, (iv) are the outcomes generalisable.”

**Modification Description:** “Thanks for the comments of the experts on this article. The introduction has been modified according to the suggestions of the external audit experts, ensuring that the justification for the study and other contents are included.”

**2.amendments**: “more discourse on the empirical approach and technique. Why is this technique used? Is it the best method that addresses the objectives of the study?”

**Modification Description:** It has been modified according to relevant requirements. Positivism Model and Data Source can be seen in detail. In the part of research methods, “DID comes from the aggregate data model of econometrics, which is a widely used econometric method in policy analysis and engineering evaluation. It is mainly applied to evaluate the impact degree of a certain event or policy in mixed cross section data set. The dual difference method does not require the experimental group and the control group to be completely consistent, there may be some differences between the two groups, allowing selection according to individual characteristics, as long as the characteristics do not change with time, which is the biggest advantage of DID. That is to partially alleviate the “selection bias” -induced endomutation. Therefore, this study chooses DID method to study RDC.” In the part of research time, due to the lag in the effect of administrative division adjustment, the RDC gradually entered the stage of strict control after 2017, and China's economy was greatly affected by the SINO-US trade war and the COVID-19 epidemic, the effect of administrative division adjustment was affected to varying degrees. So the research phase of this paper is thus identified as the cities undergoing the adjustment of administrative division of municipal districts nationwide from 1998 to 2016, which is the period when municipal district for county (county-level city) happened frequently.

**3.amendments**: “recast the Policy Recommendations”

**Modification Description:** It has been modified according to relevant requirements. See Conclusion and Discussion for details. It includes the following contents. “The adjustment of administrative divisions of municipal districts is a multi-disciplinary systematic project with dynamic, complex and comprehensive characteristics. It needs to optimize the layout according to the actual development needs of the state and local areas on the basis of following the differences and changing trends of the spatial distribution of economic factors. China's experience shows that in the process of rapid urbanization development, first, we should attach great importance to the important significance of administrative division adjustment to the governance of metropolitan areas. Timely promotion of administrative division adjustment is beneficial to reduce the cost of urbanization development and improve the governance efficiency of metropolitan areas, which is also an important experience of rapid urbanization development in the past 40 years of reform and opening up. The rest of the world should learn from this experience in light of their national conditions. The second is to promote the spatial optimization of municipal districts based on the thinking of urban agglomeration. The scientific setting of urban agglomeration scale structure can effectively release the potential of regional spatial development and improve the development efficiency of urban system. Third, the adjustment of municipal districts should focus on the development intensity and development potential of cities. It is necessary to determine whether the central urban area has urgent practical needs. Only when the development of the central urban area is restricted (under normal circumstances, the development intensity of the municipal district is above 15%) and the development intensity and development potential are both large, can it be necessary to set up new municipal districts and expand the area of the municipal district.”

**4.amendments**: “some minor grammatical and editorial corrections are required”

**Modification Description:** Modifications have been made as required, including title, text, references, formatting, grammatical and punctuation errors, etc.

**Reviewer #2:**

**1.amendments**: “the paper does not show concrete policy recommendations”

**Modification Description:** It has been modified as required.

**2.amendments**: “the dataset is old, not consider relevant factors that may contribute to policy development”

**Modification Description:** It has been explained in the paper. The adjustment of administrative division itself is a policy factor. This paper mainly discusses the effect of the policy on economic and social development.

**3.amendments**: “the author did not test for flexible models. True effects to account for the special characteristics of each administrative unit”

**Modification Description:** This paper has considered the difference of policy effects under different city sizes, different distribution regions and different development intensities. Other factors will be further detailed and discussed in future studies.

**Opinions of the editorial Department:**

**1.amendments**: “Please ensure that your manuscript meets PLOS ONE's style requirements, including those for file naming.”

**Modification Description:** It has been modified as required.

**2.amendments**: Questions about financial Disclosure

**Modification Description:** It has been explained in the article and in the cover letter.

**3.amendments**: ORCID iD matters

**Modification Description:** It has been Modified and perfected. https://orcid.org/ 0000-0001-7248-8891

**4.amendments**: “We note you have included a table to which you do not refer in the text of your manuscript. Please ensure that you refer to Tables 1, 2, 3, 4 and 6 in your text; if accepted, production will need this reference to link the reader to the Table.”

**Modification Description:** It has been modified in the paper, and each table has been marked in the paper.

**5.amendments**: Problems with references

**Modification Description:** It has been revised as required, systematic review of relevant studies has been carried out, and the number of references has been increased to 41.

Finally, I would like to thank the reviewers and editorial department for their valuable comments.
