# Supplementary material for: Economic effects of conversion from county (or county-level city) to municipal district in China
Source: PLoS One. 2022 Sep 12;17(9):e0272267. doi: 10.1371/journal.pone.0272267 (PMC9467364; doi:10.1371/journal.pone.0272267)
Supplement: Attachment — Submitted filename: Response to Reviewers.docx [file pone.0272267.s003.docx]

**Revision notes**

Dear Editor:

Thanks to the editorial teachers and external audit experts put forward valuable opinions, so that this paper benefited a lot. According to the comments and suggestions of reviewers, this paper is modified as follows:

**Reviewer #1:**

**amendments**: “All comments have been addressed.”

**Modification Description:** “The article was further proofread and revised.”

**Reviewer #2:**

**1.amendments**: “The document uses old dataset that results in unclear policy implications.”

**Modification Description:** The article has made relevant explanation. The reasons for using 1998-2016 as the research period mainly include: first, in 1997, China suspended the work of withdrawing counties and establishing cities, and the number of withdrawing counties and cities began to increase greatly; Second, after 2017, the withdrawal of counties (cities) into districts began to be gradually tightened, and gradually entered the "stage of strict control"; Third, since 2019, due to the impact of COVID-19, the economic situation has been stagnant, which has had a certain impact on the adjustment effect of administrative divisions; Fourth, referring to relevant literature, because the adjustment effect appears with a certain lag, most of the data in the past period of time is not used for analysis. So the research phase of this paper is thus identified as the cities undergoing the adjustment of administrative division of municipal districts nationwide from 1998 to 2016, which is the period when municipal district for county (county-level city) happened frequently.

**2.amendments**: “The paper does not compare the current situation with estimated results”

**Modification Description:** This article has been modified as required. In the empirical research part, the relevant cases of Zhengzhou and Harbin are added to prove the relevant estimation results with the actual situation. “When the city is in a state of rapid development, urban renewal speed will be accelerated, the existing city capital (such as infrastructure) depreciation is forced to shorten time, leading to urban renovation costs (switching costs) has increased dramatically, and fast for the development of the economy and municipal district area of the city is too small, will increase speed of development cost. Zhengzhou in Henan Province, for example, covers an area of only 1010 square kilometers, and its development intensity is close to 50%. As the development space of the central urban area is becoming increasingly tight, which is not good for the spatial layout of industries and public services, the high density of facilities leads to the problem that many newly built facilities need to be removed in the urban renewal process, such as short-lived bus stations, short-lived overpasses and short-lived public toilets. Another example is Harbin, which in recent years has promoted a large number of county (city) reform to establish districts, resulting in a municipal area of more than 10,000 square kilometers. Due to the urban management system of municipal districts, there is a large number of agricultural population, and counties and county-level cities can enjoy poverty alleviation and agricultural benefit policies such as subsidies for livestock breeding and agricultural machinery purchase, but municipal districts cannot enjoy them. This makes such municipal district public service levels cannot be compared with the central city, economic development and not equal to counties and county-level cities, even in accordance with the actual demand cannot enjoy benefit farming policy, one, two, three industry development have encountered difficulties, caused by a slowing economy, low level of urban construction and population agglomeration problems such as weak, This is also an important reason for Harbin's economic slowdown in recent years.This shows that the development intensity of municipal districts should be taken into full consideration when promoting the withdrawal of counties (cities) into districts, as either too early or too late is not conducive to the improvement of economic and social benefits.”

**3.amendments**: “There is no discussion made with the literature with the results.”

**Modification Description:** This article has been modified as required. It is mainly reflected in the basic regression results, and relevant literature is added for comparative analysis. “The analysis results are consistent with previous studies[29,42].” In addition, countermeasures and suggestions were modified in this study.

**Opinions of the editorial Department:**

**amendments**: “Any changes to the reference list should be mentioned in the rebuttal letter that accompanies your revised manuscript.”

**Modification Description:** It has been modified as required. Compared with the previous version, 25 references are deleted and 8 references 32-38 are added.

Finally, I would like to thank the reviewers and editorial department for their valuable comments.
